# Integrating Patient-Centered Electronic Health Record Communication Training into Resident Onboarding: Curriculum Development and Post-Implementation Survey Among Housestaff

**DOI:** 10.2196/mededu.8976

**Published:** 2018-01-04

**Authors:** Maria Alcocer Alkureishi, Wei Wei Lee, Sandra Webb, Vineet Arora

**Affiliations:** ^1^ Department of Academic Pediatrics University of Chicago Chicago, IL United States; ^2^ Department of Medicine University of Chicago Chicago, IL United States; ^3^ Department of Clinical Information Systems University of Chicago Chicago, IL United States

**Keywords:** electronic health records, EHR, patient-doctor relationship, communication

## Abstract

**Background:**

Electronic health record (EHR) use can enhance or undermine the ability of providers to deliver effective, humanistic patient-centered care. Given patient-centered care has been found to positively impact patient health outcomes, it is critical to provide formal education on patient-centered EHR communication skills. Unfortunately, despite increasing worldwide EHR adoption, few institutions educate trainees on EHR communication best practices.

**Objective:**

The goal of this research was to develop and deliver mandatory patient-centered EHR training to all incoming housestaff at the University of Chicago.

**Methods:**

We developed a brief patient-centered EHR use curriculum highlighting best practices based on a literature search. Training was embedded into required EHR onboarding for all incoming housestaff (interns, residents, and fellows) at the University of Chicago in 2015 and was delivered by institutional Clinical Applications Trainers. An 11-item posttraining survey consisting of ten 5-point Likert scale questions and 1 open-ended question was administered. Responses at the high end of the scale were grouped to dichotomize data.

**Results:**

All 158 of the incoming 2015 postgraduate trainees participated in training and completed surveys (158/158, 100.0%). Just over half (86/158, 54.4%) were interns and the remaining were residents and fellows (72/158, 45.6%). One-fifth of respondents (32/158, 20.2%) were primary care trainees (defined as internal medicine, pediatric, and medicine-pediatric trainees), and the remaining 79.7% (126/158) were surgical or specialty trainees. Self-perceived pre- versus posttraining knowledge of barriers, best practices, and ability to implement patient-centered EHR skills significantly increased (3.1 vs 3.9, *P*<.001 for all). Most felt training was effective (90.5%), should be required (86.7%), and would change future practice as a result (70.9%). The only significant difference between intern and resident/fellow responses was prior knowledge of patient-centered EHR use barriers; interns endorsed higher prior knowledge than resident peers (3.27 vs 2.94 respectively, *P*=.03). Response comparison of specialty or surgical trainees (n=126) to primary care trainees (n=32) showed no significant differences in prior knowledge of barriers (3.09 vs 3.22, *P*=.50), of best practices (3.08 vs 2.94, *P*=.37), or prior ability to implement best practices (3.11 vs 2.84, *P*=.15). Primary care trainees had larger increases posttraining than surgical/specialty peers in knowledge of barriers (0.8 vs 0.7, *P*=.62), best practices (1.1 vs 0.8, *P*=.08), and ability to implement best practices (1.1 vs 0.7, *P*=.07), although none reached statistical significance. Primary care trainees also rated training as more effective (4.34 vs 4.09, *P*=.03) and felt training should be required (4.34 vs 4.09, *P*=.10) and would change their future practice as a result (4.13 vs 3.73, *P*=.02).

**Conclusions:**

Embedding EHR communication skills training into required institutional EHR training is a novel and effective way to teach key EHR skills to trainees. Such training may help ground trainees in best practices and contribute to cultivating an institutional culture of humanistic, patient-centered EHR use.

## Introduction

Electronic health record (EHR) use during clinical encounters is becoming the norm worldwide [1]. Since 2006, use of EHRs by US physicians increased by over 160% with 78% of office-based physicians and 59% of hospitals having adopted an EHR by 2013 [2]. That number has only increased with time, partly due to the financial incentives put in place by the Health Information Technology for Economic and Clinical Health Act of 2009, which calls for not only expanded use of EHRs but also increased engagement with patients and families with the EHR [3].

Given their ubiquity in practice, it is important for providers to be aware of how EHR use in clinical encounters can alter the patient-provider interaction. Studies looking at the impact of EHR use on the patient-doctor relationship and communication have identified physician behaviors that may negatively impact the patient-doctor relationship; for example, typing during sensitive discussions and low rates of screen sharing can lead to a decrease in transparency of provider actions and patient confusion or distrust as to what their provider is doing on the computer [4,5]. As a result, patient satisfaction with the interaction and the perception of their overall quality of care may be decreased [4-11]. Similarly, best practices for patient-physician EHR interactions exist, and research shows that when EHRs are integrated in a patient-centered manner, physicians can foster patient activation and engagement by improving communication, enhancing patient understanding of their medical conditions, and promoting increased shared decision making [1,4,12,13].

Patient-centered care has been found to positively impact a number of measures such as patient compliance, satisfaction, and health outcomes [14]. Given these important benefits, mandates such as the World Health Organization’s Global Strategy on People-Centered and Integrated Health Services have called for improved people-centered care that empowers, educates, and engages individuals and incorporates technology in an efficient and effective manner [15]. In the United States, the American Medical Association and Accreditation Council for Graduate Medical Education also recommend educating trainee physicians on these best practice behaviors [16,17] in order to ground them in best practices and promote a lifelong practice of effective, patient-centered care.

Despite the existence of these best practices and mandates, few trainees receive training on EHR communication skills. Challenges related to providing this training include difficulty in reaching trainees across different specialties, lack of time in an already crowded curriculum, lack of a patient-centered EHR use curriculum, and lack of trained educators to provide the instruction [18]. Without formal education on best practices, trainees may pick up bad habits and develop negative “workaround behaviors,” informal practices designed to help them coordinate their day’s work especially under conditions of time pressure and constraints [19]. Although these workarounds may help trainees get through the tasks of their day, they are generally suboptimal and can negatively influence the delivery, safety, effectiveness, and efficiency of care [19]. As such, appropriately using the EHR and information technology to not only manage the process of patient care but to also engage, communicate with, educate, and empower patients is an essential redesign of clinical practice today, and it is critical trainees learn best practice behaviors early on [18].

To address the need for instruction as well as the various obstacles generally encountered in providing training, we formed a novel partnership with our institutional EHR trainers and embedded an EHR use communication curriculum into the existing required EHR onboarding process for all incoming housestaff at the University of Chicago.

## Methods

After conducting a literature review of best practices for patient-centered communication use, we developed a comprehensive patient-centered EHR use curriculum for medical students at the Pritzker School of Medicine as part of the second-year clinical skills course [20,21]. This curriculum consisted of a 1-hour interactive lecture addressing the impact of EHR use on patient-provider communication and summarized best practices using the HUMAN LEVEL mnemonic (ie, “Honor the golden minute” to elicit patients’ concerns before engaging the EHR) (Table 1) [7,20,22,23]. Students then participated in a group observed structured clinical examination where 1 student per group directly interacted with a standardized patient while the remaining students and a faculty member observed. Students were tasked with logging into and navigating a mock patient chart in the EHR and interacting with the standardized patient to discuss their chief concern, review prior labs, and provide counseling using the EHR in a patient-centered manner. A debrief and feedback from the faculty facilitator, standardized patient, and peer observers was conducted immediately after the exercise in order to highlight areas for improvement.

The above curriculum has successfully become an ongoing part of the Pritzker School of Medicine clinical skills course since 2013; however, it is time- and resource-intensive, thus limiting its direct application to all institutional postgraduate trainees [21]. As such, we consolidated the key themes and practical points of the training into a brief 15- to 20-minute, 8-slide curriculum on key patient-centered EHR use skills targeting postgraduate trainees. The curriculum highlighted best practices for patient-centered communication skills and included a review of the HUMAN LEVEL mnemonic (Table 1) [7,20,22,23].

**Table 1:** HUMAN LEVEL—10 tips to enhance patient-centered electronic health record use.

Letter	Tip	Explanation
H	Honor the “Golden Minute”	Make the start of the visit completely technology free. Greet the patient, start with their concerns, and establish an agenda for the visit *before* engaging technology.
U	Use the “Triangle of Trust”	Create a triangle configuration that puts you, the patient, and the computer screen at each of the three corners. This allows you to look at both the patient and screen without shifting your body position and also enables shared screen viewing.
M	Maximize patient interaction	Encourage patient interaction. Pause for questions and clarification. Allow time for questions and to verify understanding.
A	Acquaint yourself with chart	Review the chart before you enter the room to prepare, inform, and contextualize your visit.
N	Nix the screen	When discussing sensitive information, completely disengage from the electronic health record (EHR) (look at the patient, turn away from screen, take hands off keyboard, etc).
L	Let the patient look on	Share things on the screen with your patients.
E	Eye contact	Maintain eye contact with patients as much as possible. Treat patient encounters as you would a conversation with friends or family members.
V	Value the computer	Praise the benefits of the EHR and take advantage of opportunities to use technology as a tool to engage patients (pull up lab result to review together, use graphics, etc).
E	Explain what you’re doing	Be transparent about everything you do. Avoid long silences, aim for conversational EHR use by explaining what you are doing as you are doing it.
L	Log off	At the end of the visit, log off of the patient’s chart while they are still in the exam room. This reassures the patient that their medical information is secure.

The curriculum was embedded in the required 4-hour EHR ambulatory onboarding for all incoming housestaff at the University of Chicago in August 2015. One of the authors (MAA) delivered a 1-hour training to 5 institutional EHR trainers and provided individual feedback on practice presentations of the material. The curriculum was not embedded in the actual EHR itself; rather, it was embedded in the training of how to use the EHR. The training was provided in person (ie, not a webinar or via webcast) and was led by our institutional EHR trainers. Learners attended the training session in groups, and during the training each learner had a desktop computer that allowed them to explore and practice the various functional abilities of the EHR with respect to their provider role (eg, how to log in, view labs and studies, place orders, write notes).

The entire EHR training session was 4 hours in length, and our content on patient-centered EHR use training was 20 minutes in length. It consisted of 8 PowerPoint slides that highlighted barriers to and best practices for patient-centered EHR use as well as institutional documentation expectations (ie, authorship and professionalism, refraining from indiscriminate importing of labs or radiology reports, keeping notes succinct and up to date).

An 11-item survey consisting of ten 5-point Likert scale questions and 1 open-ended question was administered to all participants posttraining (Multimedia Appendix 1). Average responses are reported and for some questions, responses at the high end of the scale were grouped to dichotomize data (ie, 4=agree and 5=strongly agree were simply categorized as “agree”). Differences between intern versus resident or primary care versus specialty/surgical trainee responses were analyzed using Wilcoxon rank-sum tests, and differences in overall pre- versus posttraining responses were analyzed using Wilcoxon signed-rank tests. Differences in trainee responses in relationship to the EHR instructor delivering the content were analyzed using Kruskal-Wallis tests.

## Results

A total of 158 postgraduate trainees attended the training and completed surveys (158/158, 100%). Over half (86/158, 54.4%) were interns and the remaining were residents and fellows (72/158, 45.6%). One-fifth of respondents (32/158, 20.2%) were primary care trainees (defined as internal medicine, pediatric, and medicine-pediatric trainees), and the remaining 79.7% (126/158) were surgical or specialty trainees with representation from 27 different specialties.

Overall, trainees reported significant increases in their knowledge of barriers, best practices, and ability to implement best practice strategies (3.1 vs 3.9 for all, *P*<.001). On a 5-point Likert scale, 90.5% of respondents (143/158) either strongly agreed or agreed that the training was effective, and 86.7% (137/158) strongly agreed or agreed that it should be required for physicians and anyone interacting with patients and the EHR. A total of 70.9% of trainees (112/158) felt they planned to change their practice and how they interact with patients and the EHR as a result of the training.

Five different EHR instructors provided the training, and there was no significant difference in ratings of training effectiveness (*P*=.29), need for required training (*P*=.53), usefulness of the mnemonic (*P*=.58), and likelihood of changing practice (*P*=.43) across instructors.

When comparing responses with regard to training level, the only significant difference between intern and resident/fellow responses was prior knowledge of barriers to patient-centered EHR use, with interns endorsing higher knowledge than their more experienced peers (3.27 vs 2.94 respectively, *P*=.03 from Wilcoxon rank-sum test).

Response comparison of specialty or surgical trainees (n=126) to primary care trainees (n=32) showed no significant differences in prior knowledge of barriers (3.09 vs 3.22, *P*=.50), best practices (3.08 vs 2.94, *P*=.37), or prior ability to implement best practices (3.11 vs 2.84, *P*=.15). Primary care trainees tended to have larger increases posttraining than their surgical or specialty peers in knowledge of barriers (0.8 vs 0.7, *P*=.62), best practices (1.1 vs 0.8, *P*=.08), and ability to implement best practices (1.1 vs 0.7, *P*=.07), although none reached statistical significance. Likewise, they rated the training as more effective (4.34 vs 4.09, *P*=.03) and tended to agree more with the statements that training should be required (4.34 vs 4.09, *P*=.10) and that they would change their future practice as a result (4.13 vs 3.73, *P*=.02).

Responses to the open-ended question “What other suggestions/comments do you have about this session?” were in general quite positive. Of note, 1 trainee stated they would have preferred more expectations of what is to be documented in patient notes. Another trainee indicated they would have preferred additional practical training, stating “I would have preferred a longer hands-on portion with more exercises rather than the entire lecture portion. Doing a training that is more case-oriented with staff helping answer questions along the way would be a much more effective use of time.” Last, only 1 respondent mentioned that they had previously learned the HUMAN LEVEL mnemonic of best practices.

## Discussion

It is imperative that today’s health care trainees are able to manage the demands of the EHR while maintaining a meaningful relationship with the patient and foster patient-provider communication. Our short training resulted in measurable improvement on self-assessed patient-centered EHR use knowledge, attitude, and skills, as well as likelihood to affect future practice for incoming trainees regardless of resident and fellow levels or specialty type. It is interesting to note that interns endorsed higher prior knowledge of barriers than their more experienced peers. It may be that younger trainees are more attuned to obstacles with EHR use, whereas their more experienced peers have developed EHR workarounds and are thus less likely to endorse barriers. Whether those informally learned workarounds promote or undermine patient-centered EHR use is unclear, however, and the importance of grounding all trainees in best practices remains. Also, given primary care trainees rated training as more likely to change their future practice, there is likely a need to tailor training for specialty and surgical trainees in order to increase its effectiveness.

Based on trainee feedback, future curricular development should aim to provide further reinforcement and specific guidance in terms of expectations of what is to be documented in patient notes. Additional opportunities for a more involved practical session with documentation exercises and real-time feedback and guidance may also be beneficial in providing reinforcement of a practical skill set as trainees begin clinical practice with patients. Furthermore, additional training touchpoints using a variety of methods (visual reminders, in-person training with standardized patients, and perhaps even embedded reminders in the EHR) not just for trainees but for attendings as well may be helpful in ensuring providers of all levels remember and employ best practices in their clinical care.

Most importantly, it is critical to provide opportunities for trainees to obtain feedback about their skills with real patients. This can come in the form of direct feedback from patients themselves as well as from faculty supervisors observing their actual clinical practice. Tools such as the validated electronic Clinical Evaluation Exercise may be helpful in order to structure feedback on patient-centered EHR use behaviors and highlight areas for improvement [24].

Our novel approach of embedding communication training into required EHR onboarding was an effective and efficient way to teach housestaff these key EHR-related skills. It is essential to teach housestaff at this critical and early point in training when they are primed to learn and incorporate skills into practice. It is equally important, however, to provide additional booster curricula in order to reinforce and promote maintenance of behaviors and elicit feedback from actual patients on provider practice in order to identify areas for improvement.

Use of institutional EHR trainers, professional information technology specialists who already routinely educate physicians on EHR use, takes advantage of existing resources at most institutions. Also, the difficulty of delivering curricula to all housestaff across specialties is alleviated by embedding it in required onboarding training. Last, strategies for best practice exist and are easily teachable to trainees. Introducing these skills early on contributes to a culture in which professional patient-centered EHR use is the norm.

While our project is currently single site and relies on self-assessed survey questions, our future work aims to provide longitudinal reinforcement of these skills and feedback on EHR communication skills in real clinical encounters to provide continued feedback and training. In addition, we realize the importance of correlating changes in patient satisfaction with training and are focusing on training faculty to promote positive role-modeling and proactively shape the “hidden curriculum” of EHR use. We are also working toward informing patients of their role with the EHR, educating them about the function of the computer in their care and inviting them to become more involved with its use not only during the visit with their provider but afterward via patient portals and secure messaging systems.

Importantly, most trainees felt that although the training was short, it was effective and should be required. As a result, our curriculum has become a permanent part of the onboarding process for all incoming housestaff, and there are plans to expand our training to target attending physicians, midlevel providers, nurses, and support staff at the University of Chicago.

Patient-centered care has been found to positively impact a number of measures such as patient compliance, satisfaction, and health outcomes [13]. Given the increasing rates of EHR adoption, it is critical to provide physicians with formal education on patient-centered EHR communication skills; however, finding the time to teach these skills in crowded training programs poses a challenge. We found partnering with EHR trainers who deliver required onboarding training is a novel, timely, and effective method to facilitate training on patient-centered EHR communication strategies across a variety of residency and postresidency training programs. This curriculum capitalizes on existing EHR trainers and leverages resources in a cost-effective manner to provide training to a captive audience of diverse incoming trainees. Similar training can be easily replicated at other institutions and may help ground trainees in best practices and contribute to cultivating a culture of high-quality patient care and meaningful, humanistic patient-centered EHR use.

## Appendix

Multimedia Appendix 1 Patient-centered electronic medical record use training survey.

## Abbreviations

EHR: electronic health record
