# Supplementary material for: Integrating Patient-Centered Electronic Health Record Communication Training into Resident Onboarding: Curriculum Development and Post-Implementation Survey Among Housestaff
Source: JMIR Med Educ. 2018 Jan 4;4(1):e1. doi: 10.2196/mededu.8976 (PMC5773818; doi:10.2196/mededu.8976)
Supplement: Multimedia Appendix 1 [file mededu_v4i1e1_app1.pdf]

Appendix 1: Patient-Centered EMR Use Training Survey

1. **PRIOR** to today's training, please rate your **knowledge of barriers** to patient-centered EMR use

   ☐Very Low (1) ☐Low (2) ☐Moderate (3) ☐High (4) ☐Very High (5)

2. **PRIOR** to today's training, please rate your **knowledge of best practices** for patient-centered EMR use

   ☐Very Low (1) ☐Low (2) ☐Moderate (3) ☐High (4) ☐Very High (5)

3. **PRIOR** to today's training, please rate your **ability to implement best practices** for patient-centered EMR use

   ☐Very Low (1) ☐Low (2) ☐Moderate (3) ☐High (4) ☐Very High (5)

4. **AS A RESULT of** today's training, please rate your **knowledge of barriers** to patient-centered EMR use

   ☐Very Low (1) ☐Low (2) ☐Moderate (3) ☐High (4) ☐Very High (5)

5. **AS A RESULT of** today's training, please rate your **knowledge of best practices** for patient-centered EMR use

   ☐Very Low (1) ☐Low (2) ☐Moderate (3) ☐High (4) ☐Very High (5)

6. **AS A RESULT of** today's training, please rate your **ability to implement best practices** for patient-centered EMR use

   ☐Very Low (1) ☐Low (2) ☐Moderate (3) ☐High (4) ☐Very High (5)

7. The training was **effective**.

   ☐Strongly Disagree (1) ☐Disagree (2) ☐Neutral (3) ☐Agree (4) ☐Strongly Agree (5)

8. The training should be **required for physicians and anyone interacting with patients and the EMR**.

   ☐Strongly Disagree (1) ☐Disagree (2) ☐Neutral (3) ☐Agree (4) ☐Strongly Agree (5)

9. The HUMAN LEVEL mnemonic is a **useful framework** to improve patient-centered EMR use.

   ☐Strongly Disagree (1) ☐Disagree (2) ☐Neutral (3) ☐Agree (4) ☐Strongly Agree (5)

10. I plan to **change my practice** and how I interact with patients and the EMR **as a result** of this training

    ☐Strongly Disagree (1) ☐Disagree (2) ☐Neutral (3) ☐Agree (4) ☐Strongly Agree (5)

11. What other suggestions/comments do you have about this session? Open-Ended Response

Appendix 1 Key:

11-item online survey administered to all trainees upon completion of the 20 minute patient-centered EMR use curriculum training.
